# Supplementary material for: Bacterial bioindicators enable biological status classification along the continental Danube river
Source: Commun Biol. 2023 Aug 18;6:862. doi: 10.1038/s42003-023-05237-8 (PMC10439154; doi:10.1038/s42003-023-05237-8)
Supplement: Supplementary file 2 — Supplementary Information [file 42003_2023_5237_MOESM2_ESM.pdf]

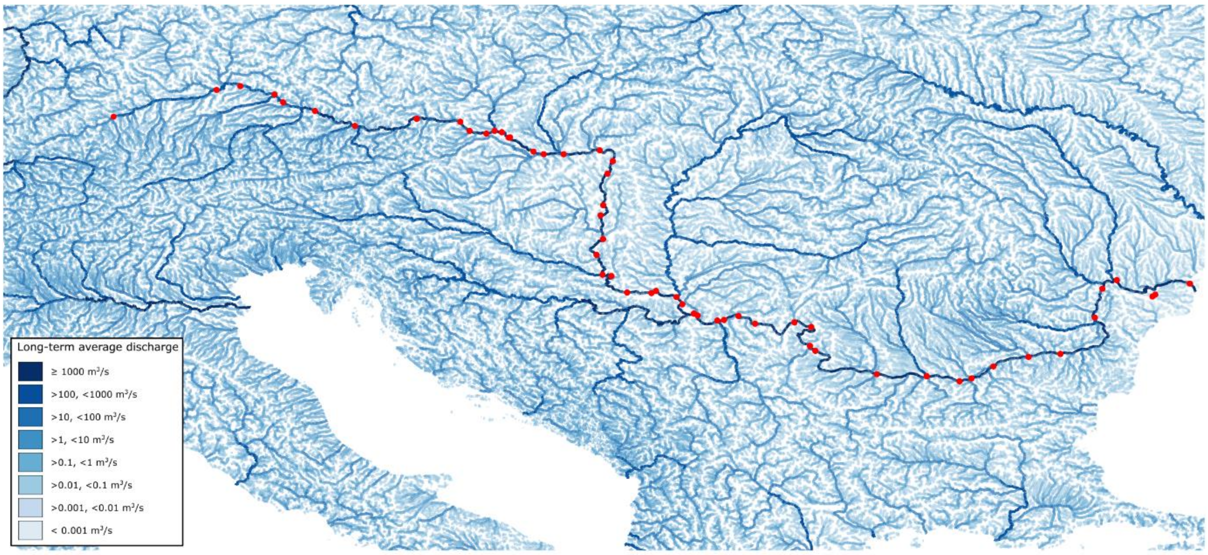

Supplementary Figure 1. Title: Joint Danube Survey 3 (JDS3) Sampling sites. **Legend:** Hydrographic map centered on the Danube catchment area with JDS3 sampling sites indicated as red dots. The color gradient represents the long-term average discharge of rivers along their flow paths.

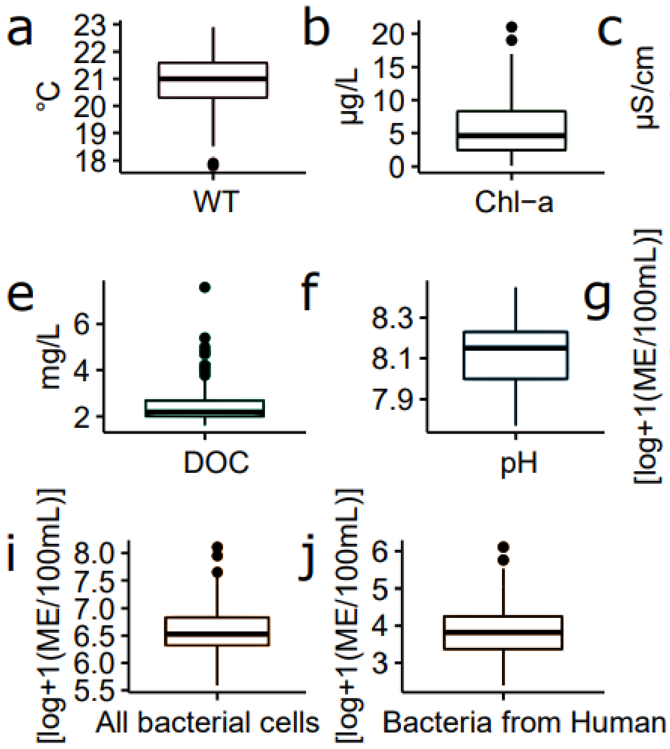

Supplementary Figure 2. **Title:** Boxplot and distribution visualization of environmental parameters (metadata) considered for the identification of bioindicator taxa related to water quality in the Redundancy Analyses. **Legend:** n=160 independent samples. The middle horizontal line represents the median, while the lower and upper lines represent the first and third quartiles interquartile range. The tip of the upper vertical line stands for the maximum of the fourth quartile, excluding outliers, as does the tip of the lower vertical line standing for the minimum of the first quartile. Dots above or below the tips of the vertical lines are outliers. a) Water temperature; b) Chlorophyll-a; c) Conductivity, d) Total nitrogen; e) Dissolved organic carbon; f) pH; g) Total coliforms; h) *Escherischia coli*; i) Counts of all bacterial cells; j) Counts of bacterial cells from human origin.

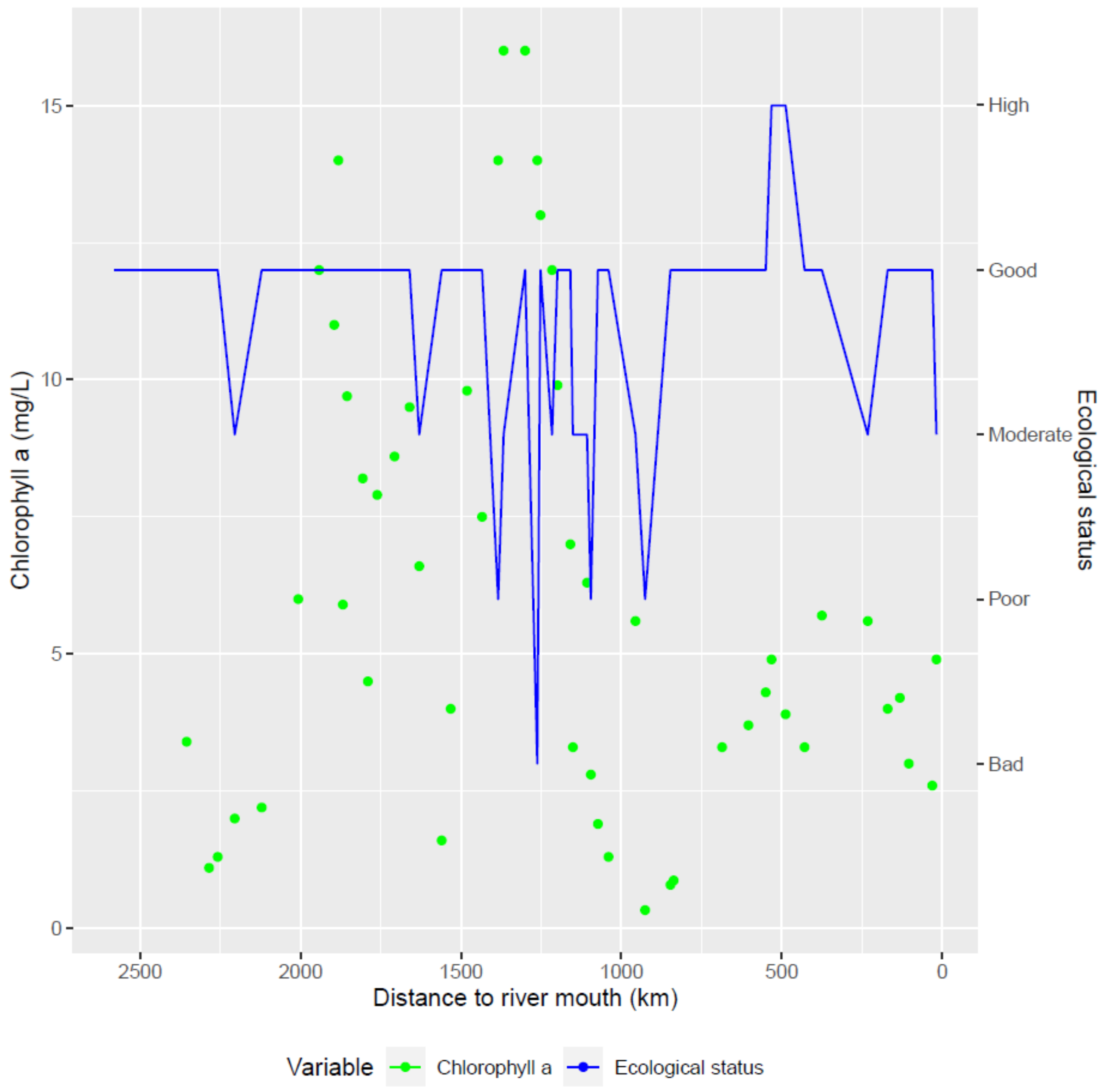

Supplementary Figure 3. Title: Ecological status and chlorophyll *a* concentration along the Danube River. **Legend:** Ecological status is presented as the continuous blue line and chlorophyll *a* concentration as the discrete green dots.

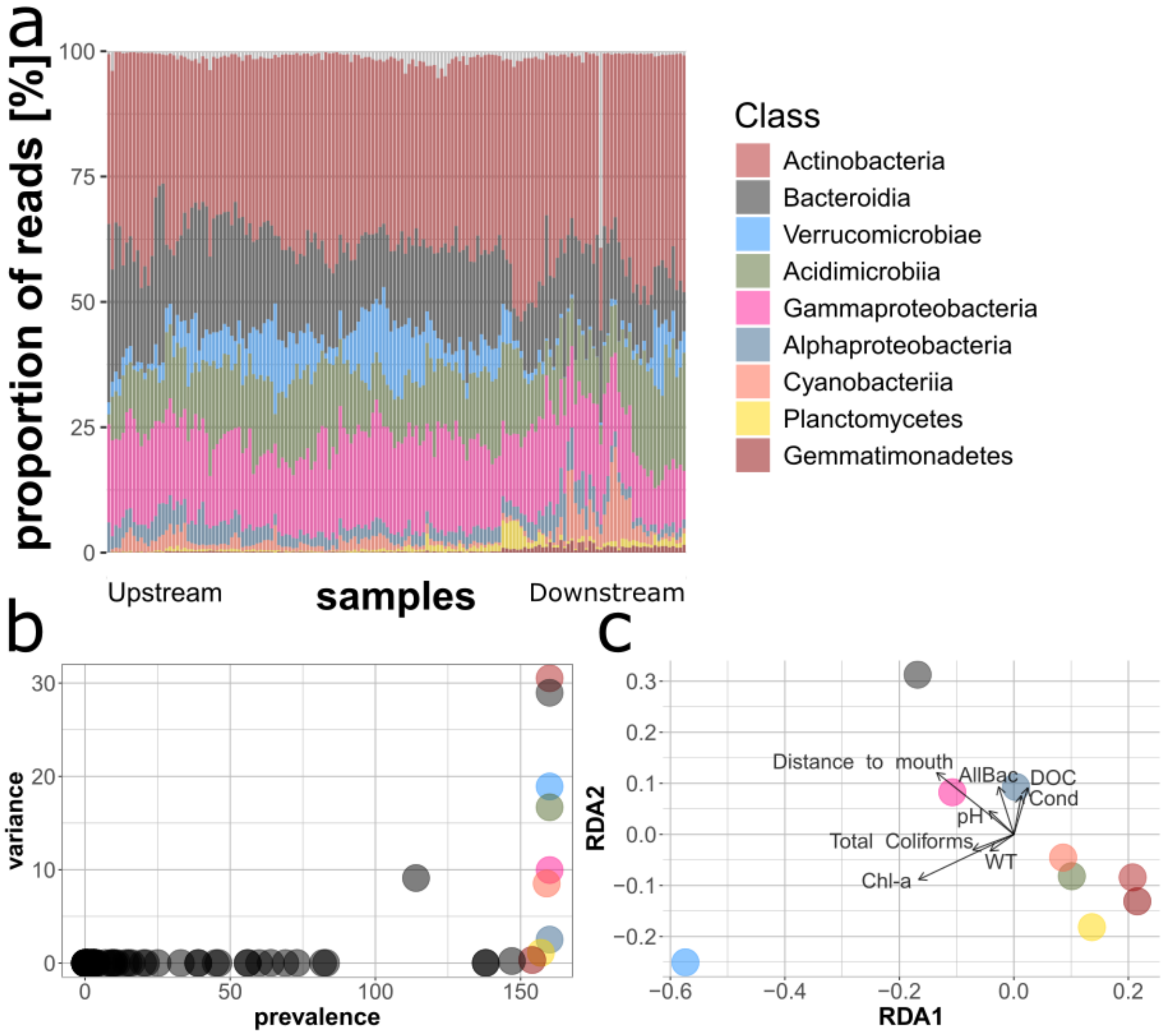

Supplementary Figure 4. Title: Visualization of the analysis on the prevalence and variance for the 9 most relevant bacterial classes for the Danube River. **Legend:** The RDA shows the relationships of each of the taxa with the environmental drivers correlating the most with the overall bacterial community patterns. (a) Dynamics of the classes among all the sampling sites. Samples are arranged from left (upstream) to right (downstream), with increasing distance from the source (b) Bacterial classes with the highest prevalence among the sampling sites and highest variance; the 9 coloured dots are the taxa most suitable as biological indicators (highlighted in the ellipses) given their broad presence and wide variance across different environments. (c) The RDA shows the relationships of each of the 9 identified classes with the environmental drivers.

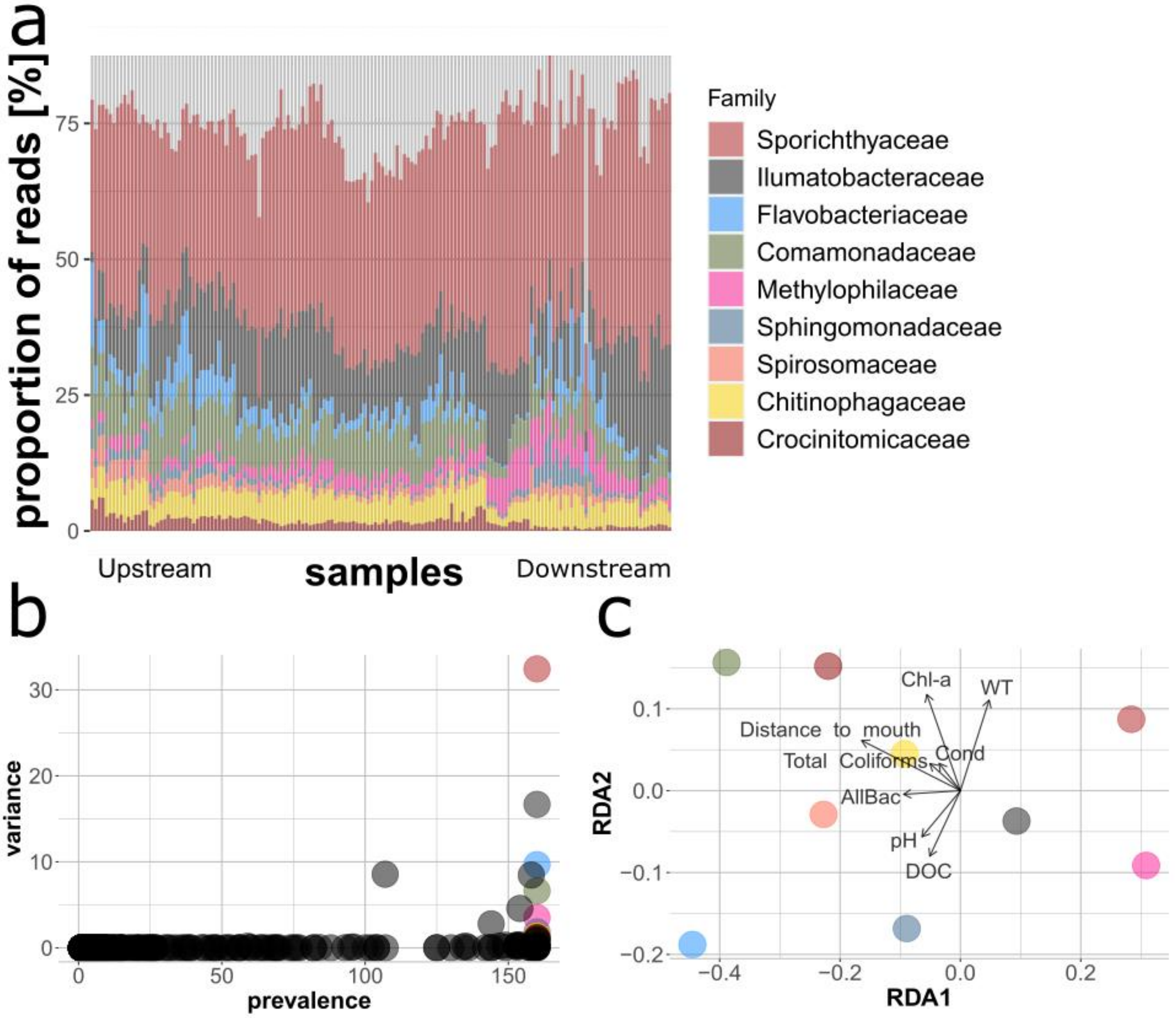

Supplementary Figure 5. Title: Visualization of the analysis on the prevalence and variance for the 9 most relevant bacterial families for the Danube River. **Legend:** The RDA shows the relationships of each of the taxa with the environmental drivers correlating the most with the overall bacterial community patterns. (a) Dynamics of the families among all the sampling sites. Samples are arranged from left (upstream) to right (downstream), with increasing distance from the source (b) Bacterial families with the highest prevalence among the sampling sites and highest variance; the 9 coloured dots are the taxa most suitable as biological indicators (highlighted in the ellipses) given their broad presence and wide variance across different environments. (c) The RDA shows the relationships of each of the 9 identified families with the environmental drivers.

Sample	Location	input	filtered	denoisedF	denoisedR	merged	nonchim
JDS01-0-M	BöfingerHalde	44572	32144	30060	30379	22028	18354
JDS02-0-L	Kelheim–gaugingstation	46899	33272	31022	31772	22686	18546
JDS02-0-M	Kelheim–gaugingstation	46527	33633	31509	32015	22792	18522
JDS02-0-R	Kelheim–gaugingstation	44717	32142	30285	30876	22472	18189
JDS03-0-L	Geislingpowerplant(upstream)	45569	31411	29234	29950	21609	17733
JDS03-0-M	Geislingpowerplant(upstream)	40867	28882	26978	27626	19359	15774
JDS03-0-R	Geislingpowerplant(upstream)	44494	31474	29734	30390	21957	18050
JDS04-0-L	Deggendorf	49958	32819	30317	31074	21523	16536
JDS04-0-M	Deggendorf	55706	37201	34760	35520	26261	21216
JDS04-0-R	Deggendorf	48179	32494	30416	30951	23240	19019
JDS05-0-L	Mühlau	46575	31920	29893	30434	22362	18045
JDS05-0-M	Mühlau	38341	23902	21967	22735	17178	13697
JDS05-0-R	Mühlau	48440	34328	32021	32803	23489	18707
JDS06-0-L	Jochenstein	40758	29047	26671	27520	18389	15626
JDS06-0-M	Jochenstein	46318	31520	29040	29716	20351	17459
JDS06-0-R	Jochenstein	49185	35013	31976	32982	21685	17783
JDS07-0-L	UpstreamdamAbwinden-Asten	42691	28882	26179	26975	16908	13153
JDS07-0-M	UpstreamdamAbwinden-Asten	38116	26222	23919	24432	16177	13449
JDS07-0-R	UpstreamdamAbwinden-Asten	37420	25240	22808	23490	14892	11803
JDS08-0-L	Oberloiben	45214	31967	28984	29801	19631	16741
JDS08-0-M	Oberloiben	48272	34539	31511	32226	21186	17700
JDS08-0-R	Oberloiben	42532	30740	28132	28908	19431	16244
JDS09-0-L	Klostemeuburg	49199	34741	32055	33065	22132	17944
JDS09-0-M	Klostemeuburg	62152	45332	41881	43018	28689	23445
JDS09-0-R	Klostemeuburg	50773	37171	34384	35452	23679	19548
JDS09-4-L	DownstreamVienna	37924	24873	21718	22837	16198	14941
JDS09-4-M	DownstreamVienna	38084	24569	21598	22563	16671	16026
JDS09-4-R	DownstreamVienna	51900	32298	29055	30970	22558	19280
JDS10-0-L	Wildungsmauer	54666	36781	33978	35469	24237	19665
JDS10-0-M	Wildungsmauer	40168	28022	25534	26466	17213	13973
JDS10-0-R	Wildungsmauer	48777	35672	32600	33772	21751	17410
JDS11-0-L	UpstreamMorava(Hainburg)	51378	36722	33779	34678	23670	19723
JDS11-0-M	UpstreamMorava(Hainburg)	56398	40720	36926	38176	25662	21302
JDS11-0-R	UpstreamMorava(Hainburg)	52088	37614	34157	35404	22932	18897
JDS13-0-L	Bratislava	43576	29885	27274	28348	18248	15424
JDS13-0-M	Bratislava	46998	33751	30527	31471	20705	16951
JDS13-0-R	Bratislava	48119	33822	30666	31766	20353	16804
JDS14-0-L	Gabcikovoresevoir	42440	29797	27233	28008	18682	15270
JDS14-0-M	Gabcikovoresevoir	43331	29800	26675	28473	18076	14916
JDS14-0-R	Gabcikovoresevoir	41182	28650	26098	27415	17951	14798
JDS15-0-L	Medvedov/Medve	42760	25828	23126	24571	17858	15690
JDS15-0-M	Medvedov/Medve	45883	31443	28792	30002	19856	16750
JDS15-0-R	Medvedov/Medve	44873	30956	28092	29051	19218	16090
JDS17-0-L	KlizskaNema	39894	26456	24240	25258	16806	13850
JDS17-0-M	KlizskaNema	44474	29909	27714	28605	19059	16039
JDS17-0-R	KlizskaNema	46396	32289	29171	30764	20151	16993
JDS19-0-L	Iza/Szony	43447	30382	28186	28779	20902	17272
JDS19-0-M	Iza/Szony	46420	32691	27431	29084	19205	16027
JDS19-0-R	Iza/Szony	42385	30280	32590	33732	23838	19626
JDS20-0-L	Szob	47542	35114	32827	33967	22969	18116
JDS20-0-M	Szob	49824	35501	29861	30904	21957	17712
JDS20-0-R	Szob	46532	32157	28845	29611	20911	16074
JDS21-0-L	Budapestupstream-MegyeriBridge	43889	30782	29161	30205	20701	16652
JDS21-0-M	Budapestupstream-MegyeriBridge	45163	31362	30436	31478	21969	17156
JDS21-0-R	Budapestupstream-MegyeriBridge	45108	32492	27170	27648	19840	16190
JDS22-0-L	Budapestdownstream-M0bridge	55517	37829	57715	58239	41172	32206
JDS22-0-M	Budapestdownstream-M0bridge	50336	33753	37765	38808	27815	21547
JDS22-0-R	Budapestdownstream-M0bridge	43548	29811	28260	28927	19960	15712
JDS24-0-L	Dunafoldvar	45586	30211	29858	30583	20148	16545
JDS24-0-M	Dunafoldvar	47204	32522	31076	32189	21387	17762
JDS24-0-R	Dunafoldvar	45755	30183	26575	27204	18157	14599
JDS25-0-L	Paks	44373	28011	25585	26524	20408	17452
JDS25-0-M	Paks	42140	26096	22593	23588	17631	15237
JDS25-0-R	Paks	37363	22887	21023	21842	16767	13904
JDS26-0-L	Baja	47154	33863	30976	31767	21089	17055
JDS26-0-M	Baja	41683	28739	20439	21190	14035	11687
JDS26-0-R	Baja	47101	33696	26762	27835	18353	15139
JDS27-0-L	Hercegszanto	33502	22672	34830	36091	24579	20154
JDS27-0-M	Hercegszanto	42038	29442	26139	27288	18329	15666
JDS27-0-R	Hercegszanto	52912	37851	29596	30954	20144	16976
JDS28-0-L	UpstreamDrava	41000	28818	40364	42527	28099	22870
JDS28-0-M	UpstreamDrava	46096	32537	39051	40989	27247	22467
JDS28-0-R	UpstreamDrava	60932	42978	29897	30152	21982	17374
JDS30-0-L	DownstreamDrava(Erdut/Bogojevo)	46956	34671	30031	31269	20519	16865
JDS30-0-M	DownstreamDrava(Erdut/Bogojevo)	49550	35752	25366	26557	16168	13316
JDS30-0-R	DownstreamDrava(Erdut/Bogojevo)	47633	33977	29688	30434	19894	17057
JDS31-0-L	Ilok/BackaPalanka	40413	28701	27156	28185	18823	16162
JDS31-0-M	Ilok/BackaPalanka	46111	33084	28840	29420	19101	15676
JDS31-0-R	Ilok/BackaPalanka	47282	33553	27135	28151	18883	16416
JDS32-0-L	UpstreamNovi-Sad	42264	29891	33768	34915	21982	17982
JDS32-0-M	UpstreamNovi-Sad	44645	32194	24931	26058	16254	12693
JDS32-0-R	UpstreamNovi-Sad	41301	29771	32796	33973	21255	17535

Sample	Location	input	filtered	denoisedF	denoisedR	merged	nonchim
JDS33-0-L	DownstreamNovi-Sad	51343	37449	30077	31110	20085	16500
JDS33-0-M	DownstreamNovi-Sad	46050	33130	27243	28277	17813	14995
JDS33-0-R	DownstreamNovi-Sad	47562	33903	35161	36233	22636	18223
JDS34-0-L	UpstreamTisa(StariSlankamen)	58976	42415	25665	26697	16892	14352
JDS34-0-M	UpstreamTisa(StariSlankamen)	42256	28699	28451	29334	19667	16053
JDS34-0-R	UpstreamTisa(StariSlankamen)	40137	27806	33221	34245	23321	18814
JDS36-0-L	DownstreamTisa/UpstreamSava(Belegis)	54892	39195	30459	31147	22694	17436
JDS36-0-M	DownstreamTisa/UpstreamSava(Belegis)	41155	28099	27478	28245	20271	15997
JDS36-0-R	DownstreamTisa/UpstreamSava(Belegis)	50317	34738	26814	27414	19794	15411
JDS38-0-L	UpstreamPancevo/DownstreamSava	49539	34547	28023	28759	17634	14080
JDS38-0-M	UpstreamPancevo/DownstreamSava	47659	33977	32662	33381	22065	17383
JDS38-0-R	UpstreamPancevo/DownstreamSava	45144	30029	24887	25366	16870	13129
JDS39-0-L	DownstreamPancevo	50414	35321	24843	25069	16132	12521
JDS39-0-M	DownstreamPancevo	39139	26557	27303	27575	18225	14403
JDS39-0-R	DownstreamPancevo	44155	30499	27350	27672	17749	13192
JDS40-0-L	UpstreamVelikaMorava	39606	27032	29301	30286	19978	15245
JDS40-0-M	UpstreamVelikaMorava	43039	29366	26334	26949	18100	14515
JDS40-0-R	UpstreamVelikaMorava	46746	31655	36578	36955	25623	19968
JDS42-0-L	DownstreamVelikaMorava	55615	39062	30557	31070	21359	16023
JDS42-0-M	DownstreamVelikaMorava	51071	35576	30968	31606	21034	15951
JDS42-0-R	DownstreamVelikaMorava	47712	32512	29709	30524	20525	15224
JDS43-0-L	BanatskaPalanka/Bazias	48124	32710	35821	36851	25636	19866
JDS43-0-M	BanatskaPalanka/Bazias	49501	33000	32187	33107	22643	17366
JDS43-0-R	BanatskaPalanka/Bazias	46470	31802	30540	31557	21116	16545
JDS44-0-L	Irongatereservoir(Golubac/Koronin)	54628	38467	34131	35301	23644	19166
JDS44-0-M	Irongatereservoir(Golubac/Koronin)	48938	34446	32323	33110	22761	17241
JDS44-0-R	Irongatereservoir(Golubac/Koronin)	48290	33039	29227	30025	20066	14680
JDS45-0-L	Irongatereservoir(Tekija/Orsova)	53864	36992	34064	35004	25639	19630
JDS45-0-M	Irongatereservoir(Tekija/Orsova)	50053	34491	28939	29791	21382	16313
JDS45-0-R	Irongatereservoir(Tekija/Orsova)	45673	31255	26749	27318	19582	14916
JDS46-0-L	Vrbica/Simijan	61354	36488	33131	34989	26421	21172
JDS46-0-M	Vrbica/Simijan	52873	32857	29869	30916	24301	18366
JDS46-0-R	Vrbica/Simijan	40942	24735	22732	23746	18110	13784
JDS47-0-L	UpstreamTimok(Rudujevac/Gruia)	53625	36155	32017	32831	22715	18409
JDS47-0-M	UpstreamTimok(Rudujevac/Gruia)	45290	30738	26563	27204	19367	14353
JDS47-0-R	UpstreamTimok(Rudujevac/Gruia)	51370	34090	28340	29049	20258	15147
JDS49-0-L	Pristol/NovoSeloHarbour	45231	30580	30755	31882	21703	16544
JDS49-0-M	Pristol/NovoSeloHarbour	42138	28505	32875	33670	23914	19429
JDS49-0-R	Pristol/NovoSeloHarbour	47552	32221	32731	33224	24949	19983
JDS50-0-L	DownstreamKozloduy	51551	35292	25293	25902	19284	15476
JDS50-0-M	DownstreamKozloduy	50635	34323	28117	28873	21233	16393
JDS50-0-R	DownstreamKozloduy	41077	26769	38764	39611	28804	22861
JDS52-0-L	DownstreamOlt	58748	41903	35321	36033	27547	20686
JDS52-0-M	DownstreamOlt	42862	29546	32049	32969	23168	17869
JDS52-0-R	DownstreamOlt	44664	29842	29519	30058	22428	18588
JDS53-0-L	DownstreamZimnicea/Svishtov	49680	34105	30896	31662	24385	18958
JDS53-0-M	DownstreamZimnicea/Svishtov	45460	30980	27563	28122	20548	14975
JDS53-0-R	DownstreamZimnicea/Svishtov	49337	32547	26917	27810	19033	15476
JDS55-0-L	DownstreamJantra	40905	25007	22536	23787	17364	15337
JDS55-0-M	DownstreamJantra	42111	29073	29943	30575	22197	17808
JDS55-0-R	DownstreamJantra	41591	28007	27928	28564	21208	16696
JDS57-0-L	DownstreamRuse/Giurgiu	45704	30494	28669	29263	19891	17325
JDS57-0-M	DownstreamRuse/Giurgiu	46751	31578	31580	32530	22768	18815
JDS57-0-R	DownstreamRuse/Giurgiu	45028	29544	31328	32541	21797	17756
JDS59-0-L	DownstreamArges,Oltenita	48487	34260	26563	27074	19305	15445
JDS59-0-M	DownstreamArges,Oltenita	62389	45250	51811	52968	38251	28936
JDS59-0-R	DownstreamArges,Oltenita	42503	28108	24939	25569	18719	13075
JDS60-0-L	Chiciu/Silistra	43200	30973	37210	37878	26927	20360
JDS60-0-M	Chiciu/Silistra	77308	54763	33946	34511	24843	19374
JDS60-0-R	Chiciu/Silistra	37246	26335	27676	28227	20169	15192
JDS61-0-L	Giurgeni	54324	39511	31092	31696	22918	17392
JDS61-0-M	Giurgeni	51773	36178	27829	28412	20614	15374
JDS61-0-R	Giurgeni	42684	29468	28298	28752	21286	16569
JDS62-0-L	Braila	48970	33056	37192	37914	27893	20234
JDS62-0-M	Braila	44866	29956	26636	27352	19951	14795
JDS62-0-R	Braila	44003	28956	38721	39363	30104	21546
JDS65-0-L	Reni	42875	28568	29060	29731	21529	17188
JDS65-0-M	Reni	50652	33885	31243	31927	23753	19391
JDS65-0-R	Reni	58116	40704	27988	28565	20468	15615
JDS66-0-L	Vilkova-Chiliaarm/Kiliaarm	44102	31461	26364	26932	19587	15501
JDS66-0-M	Vilkova-Chiliaarm/Kiliaarm	51920	33353	30875	31810	25910	24096
JDS66-0-R	Vilkova-Chiliaarm/Kiliaarm	49587	33701	32868	33551	24666	19325
JDS67-0-L	Sulina-Sulinaarm	43685	29948	32911	33491	24864	19019
JDS67-0-M	Sulina-Sulinaarm	41650	28317	23994	24589	17817	14049
JDS67-0-R	Sulina-Sulinaarm	52415	34972	29893	30434	22362	18045
JDS68-0-L	Sf.Gheorghe-Sf.Gheorghearm	50809	34982	31689	32276	24062	19203
JDS68-0-M	Sf.Gheorghe-Sf.Gheorghearm	40106	25821	30060	30379	22028	18354
JDS68-0-R	Sf.Gheorghe-Sf.Gheorghearm	50880	30770	27855	28491	23197	19121

Supplementary Table 1. Sample and sequence data overview. White/grey color coding is provided as a visual aid to group samples by sampling sites, i.e. right, left and center transects of site 2 in white; right, left and center transects of site 3 in grey; repeat for remaining sites.
