# Supplementary material for: Bacterial bioindicators enable biological status classification along the continental Danube river
Source: Commun Biol. 2023 Aug 18;6:862. doi: 10.1038/s42003-023-05237-8 (PMC10439154; doi:10.1038/s42003-023-05237-8)
Supplement: Supplementary file 3 — Description of Additional Supplementary Files [file 42003_2023_5237_MOESM3_ESM.pdf]

Description of Additional Supplementary Files

File name: Supplementary Data 1
Description: The source data behind the histogram on Figure 1.

File name: Supplementary Data 2
Description: The source data behind the histogram on Figure 2.

File name: Supplementary Data 3
Description: The source data behind the histogram on Figure 5.
