# Supplementary material for: Bacterial bioindicators enable biological status classification along the continental Danube river
Source: Commun Biol. 2023 Aug 18;6:862. doi: 10.1038/s42003-023-05237-8 (PMC10439154; doi:10.1038/s42003-023-05237-8)
Supplement: Supplementary file 7 — Reporting Summary [file 42003_2023_5237_MOESM7_ESM.pdf]

nature portfolio

Corresponding author(s): Alexander Eiler

Last updated by author(s): Jul 18, 2023

Reporting Summary

Nature Portfolio wishes to improve the reproducibility of the work that we publish. This form provides structure for consistency and transparency in reporting. For further information on Nature Portfolio policies, see our Editorial Policies and the Editorial Policy Checklist.

Statistics

For all statistical analyses, confirm that the following items are present in the figure legend, table legend, main text, or Methods section.

n/a	Confirmed	
☐	☒	The exact sample size (*n*) for each experimental group/condition, given as a discrete number and unit of measurement
☐	☒	A statement on whether measurements were taken from distinct samples or whether the same sample was measured repeatedly
☒	☐	The statistical test(s) used AND whether they are one- or two-sided<br>*Only common tests should be described solely by name; describe more complex techniques in the Methods section.*
☐	☒	A description of all covariates tested
☒	☐	A description of any assumptions or corrections, such as tests of normality and adjustment for multiple comparisons
☒	☐	A full description of the statistical parameters including central tendency (e.g. means) or other basic estimates (e.g. regression coefficient) AND variation (e.g. standard deviation) or associated estimates of uncertainty (e.g. confidence intervals)
☒	☐	For null hypothesis testing, the test statistic (e.g. *F*, *t*, *r*) with confidence intervals, effect sizes, degrees of freedom and *P* value noted<br>*Give P values as exact values whenever suitable.*
☒	☐	For Bayesian analysis, information on the choice of priors and Markov chain Monte Carlo settings
☒	☐	For hierarchical and complex designs, identification of the appropriate level for tests and full reporting of outcomes
☒	☐	Estimates of effect sizes (e.g. Cohen's *d*, Pearson's *r*), indicating how they were calculated

Our web collection on statistics for biologists contains articles on many of the points above.

Software and code

Policy information about availability of computer code

Data collection	No software was used.
Data analysis	Sequencing data processing was performed in R 3.6.2 using dada2 and phyloseq libraries. Supervised learning with XG boost was carried out with the sckikit-learn implementation of the latter in Python 3.8.8. Multivariate, network analyses and Random Forest unsupervised learning were performed in R 3.6.2 using vegan, igraph and randomForest libraries. Custom code used for the manuscript was deposited in a GitHub repository: https://github.com/alper1976/danube_indicators (DOI: 10.5072/zenodo.1222217).

For manuscripts utilizing custom algorithms or software that are central to the research but not yet described in published literature, software must be made available to editors and reviewers. We strongly encourage code deposition in a community repository (e.g. GitHub). See the Nature Portfolio guidelines for submitting code & software for further information.

Data

Policy information about availability of data

All manuscripts must include a data availability statement. This statement should provide the following information, where applicable:

- Accession codes, unique identifiers, or web links for publicly available datasets
- A description of any restrictions on data availability
- For clinical datasets or third party data, please ensure that the statement adheres to our policy

All data, sampling methods as well as analytical methods are publicly available via the official website of the International Commission for the Protection of the

Danube River (ICPDR; http://www.icpdr.org/wq-db/) and the final scientific report (Liška I, Wagner, F., Sengl, M., Deutsch, K., Slobodník, J. Joint Danube Survey 3: A comprehensive analysis of Danube water quality. Vienna, Austria; 2015.). Selected data from JDS3 (2013) were published previously in several studies (Liška I, Wagner, F., Sengl, M., Deutsch, K., Slobodník, J. Joint Danube Survey 3: A comprehensive analysis of Danube water quality. Vienna, Austria; 2015., Kirschner AKT, Reischer GH, Jakwerth S, Savio D, Ixenmaier S, Toth E, et al. Multiparametric monitoring of microbial faecal pollution reveals the dominance of human contamination along the whole Danube River. Water Res. 2017 Nov;124:543–55.).
Ecological status classification compliant with the Water Framework Directive was performed from JDS3 data collected for some of the biological communities analysed along the river, among which macroinvertebrates, macrophytes, fish and phytoplankton. Different biological classification systems are used by different countries and intercalibrating the different methods remains challenging (Birk S, Willby NJ, Kelly MG, Bonne W, Borja A, Poikane S, et al. Intercalibrating classifications of ecological status: Europe's quest for common management objectives for aquatic ecosystems. Sci Total Environ. 2013 Jun;454–455:490–9.). The Saprobic Index, based on benthic macroinvertebrate communities, is one of the best-established classification systems to assess biological status in compliance with the WFD and is mainly used to assess organic pollution. This was the only classification system used consistently throughout the various countries along the Danube River and was therefore used as the reference biological classification system in our study.
Data from the ICPDR that were used in this manuscript can be found on github – https://github.com/alper1976/danube_indicators (DOI: 10.5072/zenodo.1222217).
All sequencing data is available in NCBI Sequence Read Archive under accession number PRJNA835446. Additional data can be obtained upon request from the ICPDR.

Research involving human participants, their data, or biological material

Policy information about studies with human participants or human data. See also policy information about sex, gender (identity/presentation), and sexual orientation and race, ethnicity and racism.

Reporting on sex and gender	*Use the terms sex (biological attribute) and gender (shaped by social and cultural circumstances) carefully in order to avoid confusing both terms. Indicate if findings apply to only one sex or gender; describe whether sex and gender were considered in study design; whether sex and/or gender was determined based on self-reporting or assigned and methods used. Provide in the source data disaggregated sex and gender data, where this information has been collected, and if consent has been obtained for sharing of individual-level data; provide overall numbers in this Reporting Summary. Please state if this information has not been collected. Report sex- and gender-based analyses where performed, justify reasons for lack of sex- and gender-based analysis.*
Reporting on race, ethnicity, or other socially relevant groupings	*Please specify the socially constructed or socially relevant categorization variable(s) used in your manuscript and explain why they were used. Please note that such variables should not be used as proxies for other socially constructed/relevant variables (for example, race or ethnicity should not be used as a proxy for socioeconomic status). Provide clear definitions of the relevant terms used, how they were provided (by the participants/respondents, the researchers, or third parties), and the method(s) used to classify people into the different categories (e.g. self-report, census or administrative data, social media data, etc.) Please provide details about how you controlled for confounding variables in your analyses.*
Population characteristics	*Describe the covariate-relevant population characteristics of the human research participants (e.g. age, genotypic information, past and current diagnosis and treatment categories). If you filled out the behavioural & social sciences study design questions and have nothing to add here, write "See above."*
Recruitment	*Describe how participants were recruited. Outline any potential self-selection bias or other biases that may be present and how these are likely to impact results.*
Ethics oversight	*Identify the organization(s) that approved the study protocol.*

Note that full information on the approval of the study protocol must also be provided in the manuscript.

Field-specific reporting

Please select the one below that is the best fit for your research. If you are not sure, read the appropriate sections before making your selection.

☐ Life sciences ☐ Behavioural & social sciences ☒ Ecological, evolutionary & environmental sciences

For a reference copy of the document with all sections, see nature.com/documents/nr-reporting-summary-flat.pdf

Ecological, evolutionary & environmental sciences study design

All studies must disclose on these points even when the disclosure is negative.

Study description	The study design is a spatial series where links between observations are unidirectional (upstream to downstream) and from geographic distance. There are 160 observations across 60 spatial steps.
Research sample	This study involves an existing dataset known as the Joint Danube Survey 3, produced by the International Commission for the Protection of the Danube River (ICPDR).
Sampling strategy	No sample size calculation were performed because this study is not experimental.
Data collection	For the full description of sample collection and personnel, see JDS3 full scientific report (http://www.danubesurvey.org/jds3/jds3-files/nodes/documents/jds3_final_scientific_report_1.pdf).

Timing and spatial scale	Data was collected once at 60 sites along the Danube river, from 2581 to 18 km from the river mouth, from August 13th to September 25th 2013. Three transects were used: left river bank, right river bank, river center. It was not possible to collect all samples at all sites simultaneously due to the extraordinary amount of human and material resources this would require.
Data exclusions	No data were excluded from the analyses.
Reproducibility	No experimental findings are presented in this study.
Randomization	Randomization was not used since the study design is a non-experimental spatial series.
Blinding	Blinding was not relevant in this study as it is not experimental in nature.

Did the study involve field work? ☒ Yes ☐ No

Field work, collection and transport

Field conditions	Sampling was carried out from August 13th until September 25th 2013 along the length of the Danube; field conditions can be described as Central European summer weather.
Location	60 sites were sampled along the Danube river, from 2581 to 18 km from the river mouth. See JDS3 full scientific report (http://www.danubesurvey.org/jds3/jds3-files/nodes/documents/jds3_final_scientific_report_1.pdf) for full information on location.
Access & import/export	The signatories of the Danube Declaration, adopted at the International Commission for the Protection of the Danube River (ICPDR) Ministerial Meeting in 2010, requested the ICPDR to prepare a third Joint Danube Survey (JDS3) to be held in 2013.
Disturbance	Organism and sediment collection from the river bed, organism collection from the water column.

Reporting for specific materials, systems and methods

We require information from authors about some types of materials, experimental systems and methods used in many studies. Here, indicate whether each material, system or method listed is relevant to your study. If you are not sure if a list item applies to your research, read the appropriate section before selecting a response.

Materials & experimental systems

n/a	Involved in the study	
☒	☐	Antibodies
☒	☐	Eukaryotic cell lines
☒	☐	Palaeontology and archaeology
☒	☐	Animals and other organisms
☒	☐	Clinical data
☒	☐	Dual use research of concern
☒	☐	Plants

Methods

n/a	Involved in the study	
☒	☐	ChIP-seq
☒	☐	Flow cytometry
☒	☐	MRI-based neuroimaging
